# Canonical correlation between body-posture deviations and gait disorders in children with cerebral palsy

**DOI:** 10.1371/journal.pone.0234654

**Published:** 2020-06-16

**Authors:** Andrzej Szopa, Małgorzata Domagalska-Szopa, Andrzej Siwiec, Ilona Kwiecień-Czerwieniec

**Affiliations:** 1 Department of Physiotherapy, School of Health Sciences in Katowice, Medical University of Silesia, Katowice, Poland; 2 Department of Medical Rehabilitation, School of Health Sciences in Katowice, Medical University of Silesia, Katowice, Poland; 3 Pediatrics Center John Paul II in Sosnowiec, Sosnowiec, Poland; The Ohio State University, UNITED STATES

## Abstract

Children with Cerebral Palsy (CP) show the postural constraints while standing, and gait disorders, resulting from both primary and secondary impairments of brain injury. In our previous studies, several characteristic postural and gait patterns in children with unilateral as well as with bilateral CP were defined, and the relationship between these patterns was demonstrated. The purpose of present study was to identify which features of body posture deviation during standing were strongly related to gait deviations in independently ambulatory children with CP. For this aim we explored the cross—relationship between features of body posture while standing examined by surface topography and the selected gait parameters from three-dimensional instrumented gait analysis in one hundred twenty children with cerebral palsy, aged between 7 and 13 years, who were able to walk independently. First, our study documented that that sagittal misalignment of the spine curvature was significantly related to kinematic deviations such as deviations of pelvic tilt, inadequate swing phase and knee flexion, and peak dorsiflexion in stance. Second, the study shows that the static asymmetry of pelvis and trunk was significantly associated with kinematic deviations during gait cycle such as pelvic rotation, hip abduction in swing, ROM of knee flexion, peak dorsiflexion in stance. Based on obtained results and referring to our previous findings it can be assumed that the first model of the relationship between postural deviation and gait disturbances, called ‘postural and gait complex of disorders in sagittal plane’, is related to children with bilateral CP, whereas the second model ‘postural and gait complex of disorders in coronal plane’ to children with unilateral CP. The clinical applications of this study relate to the early recognition of particular features of postural deviation using surface topography, instead of more difficult and demanding expensive tools 3-D gait analysis.

## 1. Introduction

Cerebral palsy (CP) is an impairment of postural control and manifestation of motor dysfunction of non-progressive brain damage; CP occurs early in life [1]. Although the cerebral insult is fixed and non-progressive, the primary impairments caused by the upper motor neuron syndrome can lead to the development of secondary impairments. These include inadequate growth of muscles, which can cause contractures (shortening) of muscles and tendons, bone deformities, misalignment of joints, and excessive fatigue upon movement and walking [1]. Such primary and secondary impairments are often complex, resulting in a set of persistent disorders of posture and gait [1–3].

The localisation of CP can be unilateral or bilateral. Unilateral cerebral palsy (UCP) is a subtype of CP in which the limbs on one side of the body are involved. Bilateral cerebral palsy (BCP) is a subtype in which the limbs on both sides are involved [4]. Severity of dysfunction in children with CP can best be classified according to the Gross Motor Function Classification System (GMFCS) [5]: the higher the level in the GMFCS, the more severe is the manifestation of CP. Only children at GMFCS level I–III can walk without assistance.

Children with CP show problems with positioning of the body in space, misalignments in posture while standing, and gait disorders, which result from primary and secondary impairments in CP [6–12]. The postural and gait patterns of children with CP can differ considerably. However, in our previous studies, several characteristic postural and gait patterns in children with UCP as well as those with BCP were defined, and the relationship between these patterns was demonstrated [13–16]. Those results suggested that abnormal postural patterns can be a major component of gait disorders in CP. Although scholars have highlighted the dependence of postural control on functionality [7,8], the relationship between the standing posture and walking function has rarely been investigated in children with CP [11].

Accordingly, we investigated the relationship between body-posture deviations and gait disorders in children with CP by an innovative statistical method—canonical correlation analysis (CCA) and the structural equation model. The purpose of present study was to identify the most important features of body posture deviation while standing and specific gait disorders, as well as to recognize the relationship between them in independently ambulatory children with CP.

For this aim we explored the canonical correlation between features of body posture while standing measured by surface topography and the selected gait parameters that comprise the Gillette Gait Index (GGI) in a large cohort of children with CP. The present study may aid better understanding of aspects concerning the relationship between body-posture deviations and gait disorders in this population.

## 2. Materials and methods

### 2.1. Ethical approval of the study protocol

The study protocol was approved by the Bioethics Committee of our institution (NN-013-350/I/03/09). All work was carried out in accordance with the Code of Ethics of the World Medical Association (Declaration of Helsinki). Parents/guardians provided written informed consent before enrolment of their children in this study.

### 2.2. Inclusion criteria

The inclusion criteria were patients: (i) with a diagnosis of UCP or BCP; (ii) aged ≥7 years (to minimise the prevalence of unstable gait patterns); (iii) who could walk without assistance.

### 2.3. Exclusion criteria

The exclusion criteria were patients: (i) administered pharmacological agents at the time of study; (ii) who underwent spasticity management <6 months before evaluation; (iii) who had hip dislocation or fracture of the lower limbs previously; (v) with a history of uncontrolled seizures or vestibular dysfunction; (v) who had lower-limb surgery previously; (vi) with comorbidity that could influence the gait pattern (e.g., cardiopulmonary disorders, diabetes mellitus, asthma).

### 2.4. Participants

Children with a diagnosis of spastic CP who were the outpatients of local paediatric rehabilitation centres and who met the inclusion criteria stated above were evaluated sequentially.

Of the 127 children who met the inclusion criteria, 120 children with CP (UCP, 62; BCP, 58) aged between 7 years and 13 years (age (mean ± SD) 11.0 ± 2.1 years) who could walk independently (52 females and 68 males) were included in this study. Five participants were excluded because they could not follow verbal directions during examination, and two participants were excluded because they refused to undress for examination. Participants were classified by GMFCS into two levels: I (walks without limitations; n = 68) and II (walks with limitations; n = 52) and their GMFM-88 score on average was 67.6% (49.2–97.9) [17].

### 2.5. Testing procedures

The present study is a follow-up and extension of a previous four-part series on the functional assessment of children with CP [13–16]. Our study comprised two interrelated parts: (i) surface topography of body posture; (ii) three-dimensional instrumented gait analyses (3DIGA).

#### 2.5.1 Surface topography of body posture

The group of children with CP underwent examination of surface topography based on the Moiré phenomenon (i.e. projection Moiré topography (MT). MT was used to obtain a graphical representation of the body posture for a quantitative assessment of postural patterns [18,19]. MT is an optical method used to assess deviations of the back-surface contours associated with deformities in body posture. Children were tested using MT system (CQ Elektronik System, Czernica, Poland).

A few studies assessed the accuracy of MT measurement in respect to other methods of body posture examination. The high repeatability and reproducibility of MT examination has been demonstrated by Chowańska et al. [18]. In addition, MT and radiography are highly correlated [20–22]. Moreover, use of MT for postural assessment can reduce radiation exposure in growing children.

For examination of posture, we used the same experimental method described in our previous studies for investigating body alignment in a standing position in children with UCP and those with BCP [13,15]. During MT examination, participants stood relaxed with arms alongside their trunk, barefoot, in an uncorrected and quiet stance. For MT, it was necessary to uncover the entire surface of the back and identify some anatomical landmarks: the spinous process of C_7_, spinous process of S1, acromial angle of shoulders, superior angle of the scapula, inferior angle of the scapula, and posterior superior iliac spine. Several indices were measured in our study. In the sagittal plane, the angle of trunk inclination in the sagittal plane, angle of pelvic tilt, angle of kyphosis, angle of lordosis, and the difference between the angle of kyphosis and angle of lordosis were measured. In the coronal plane, the trunk inclination index in the coronal plane, angle of vertebral lateral curvature, angle of shoulder line inclination, and angle of pelvis obliquity was measured. In the transverse plane, the angle of trunk rotation, angle of shoulder rotation, and angle of pelvic rotation were measured [13,15].

To determine inter-rater reliability (AS vs MDS), both examiners performed the analysis of the same, randomly selected set of 20 topograms (initial analysis). To determine the stability of intra-rater measure (AS vs AS and MDS vs MDS), both examiners after 48 hours performed an analysis of the same set of 20 topograms (repeated analysis). In both initial and repeated analysis, the two examiners performed the evaluations independently, blind to the allocation of topograms.

#### 2.5.2 3DIGA

3D kinematic data were collected using the Compact Measuring System for 3D Real-Time Motion Analysis based on 15 active (five triplicate) ultrasound markers using WinGait^™^ (Zebris Medizintechnik, Weitnau, Germany). Gait data were recorded as the participants walked barefoot on a treadmill (Alpha XL; Kettler, Ense, Germany). Before conducting gait analyses, several anatomical landmarks were identified with an instrumented pointer: hip joint centre; knee centre (medial and lateral femoral epicondyle); ankle rotation centre (internal, and external); forefoot landmark (between the second and third metatarsals); rear foot (heel). Before data collection, all participants could walk on the treadmill to test it out. Children walked without shoes and without assistive devices. Ultrasonic markers were attached to the skin with double-adhesive tapes and placed bilaterally. By trial-and-error, the speed of the treadmill was adjusted based on the ability of each child to ensure the most natural gait for every individual and was 2.45±0.36 km/h on average (walking speed = 0.68±0.17 m/sec; cadence = 0.85±0.18 per sec). Three trials were undertaken with 2–5 strides in each trial.

The GGI comprises 16 distinct gait parameters. The GGI was calculated (separately for each lower limb) according the procedure described by Schutte et al. and Romei and colleagues [23,24]. The 16 parameters are: (1) stance phase (expressed as the percentage of the gait cycle); (2) walking speed (normalised to leg length); (3) cadence; (4) mean pelvic tilt; (5) range of motion (ROM) of pelvic tilt; (6) mean pelvic rotation; (7) minimum hip flexion; (8) ROM of hip flexion/extension; (9) peak hip abduction in swing; (10) mean hip rotation in stance; (11) knee flexion at initial contact; (12) time to peak knee flexion in swing (expressed as the percentage of the gait cycle); (13) ROM of knee flexion; (14) peak dorsiflexion in stance; (15) peak dorsiflexion in swing; (16) mean foot progression.

### 2.6. Statistical analyses

The Intraclass Correlation Coefficient (ICC) with 95% confidence interval to evaluate the intra-observer and interobserver agreement across MT parameters were used. Absolute reliability was determined by calculating both the standard deviation of measurement errors (SEM) and the (minimal detectable change) MDC.

Originally, 11 MT indices and GGI and 16 gait parameters were defined for each participant. The mean values of MT indices from three measurements and mean values for GGI and 16 distinct gait parameters (averaged for both lower limbs) from three gait trials were used for statistical analyses. The high dimensionality of the data for postural analysis and gait analysis meant that factorial analysis had to be employed to reduce the number of variables [25]. It was assumed that only variables for which the absolute values of the factor loadings were >0.7 were associated with large values of factor loadings and carried the appropriate amount of information [25]. Therefore, these variables were considered in further analysis for canonical correlation. The relationship between the variable set MT indices (predictor set) and variable set gait kinematics (criterion set) was analysed using CCA [26]. There are several nonparametric measures of relationships based on the similarity of ranks in two variables (e.g., multiple regression, principal component analysis, multiple correspondence analysis). Nevertheless, CCA as a new technique in the clinical domain is the most appropriate procedure to investigate the relationship between two sets of variables.

## 3. Results

The intra-observer error across MT parameters (TT, ALC, PO, SHI, K, LL) was 0.82–0.90 (mean 0.86), whereas the inter-observer error was 0.78–0.86 (mean 0.82). Both values reflected excellent reliability [27]. SEM and MDC values across MT parameters (TT, ALC, PO, K, LL) comparable between observers (AS vs MD) were from 0.49° to 1.65° and 0.23° to 4.57°, respectively. SEM and MDC values comparable between repeated measurements were varying from 0.19° to 2.82° and 0.50° to 7.82°, respectively for first assessor (AS1 vs AS2) and between 0.19 to 2.90 and 0.53 to 8.03 respectively for second assessor (MD1 vs MD2).

CCA was conducted using nine postural variables (Table 1) as predictors of 12 gait variables (Table 2) to evaluate the multivariate shared relationship between the two variable sets (i.e., body-posture deviations and gait disorders in children with CP).

**Table 1:** Description of variable set Moiré topography (MT) indices (predictor set).

MT indices	Mean± SD	Median	Maximum	Minimum
TI (mm)	347.3±47.5	337.8	478.7	273.2
TT (°)	5.7±4.5	4.1	16.3	0.1
ACL (°)	-0.4±1.6	-0.5	2.8	-3.7
PT (°)	1.4±0.9	1.3	3.7	0
PO (°)	0.6±3.2	0.7	8.9	-12.0
SHI (°)	-0.2±10.0	2.0	16.1	-20.9
SHR (°)	8.8±4.7	7.9	20.9	0
LL (°)	6.4±0.6	6.1	7.2	5.8
KL (°)	-0.6±6.8	-1.4	35.0	-16.9

MT indices: trunk inclination index in the coronal plane (TI); angle of trunk inclination in the sagittal A-P plane (TT); angle of vertebral lateral curvature (ALC); angle of pelvic tilt (PT); angle of shoulder line inclination in the coronal M-L plane (SHI); angle of shoulder rotation (SHR); angle of pelvic obliquity (PO); angle of lordosis (LL); difference between angle of kyphosis and angle of lordosis (KL index).

TI refers to the magnitude of the distance measured from the midline situated within the coronal plane and a line connecting the spinous processes from C7 through S1. If C7 is anterior to S1, TI has the negative (−) value and the positive (+) value in the opposite case.

TT is the angle contained between two adjacent lines situated within the coronal plane and a line connecting the spinous processes from C7 through S1. If C7 is anterior to S1, TT value ranges from -180° to 0°; conversely, the TT value ranges from 180° to 0° in the opposite case.

ALC is the angle contained between two adjacent lines situated within the sagittal plane and a line connecting the spinous processes from C7 through S1. If the apex of the lateral curve is on the right side of the vertical line, the value of ALC ranged from 0° to 180°; if was on the left of the vertical line, ALC ranged from -180° to 0°.

PT is the angle contained between two adjacent lines as follows: a line connecting C7 with S1 and a line connecting *L*max with S1. If *L*max is anterior to a line C7-S1, PT value ranges from -180° to 0°; conversely, the PT value ranges from 180° to 0° in the opposite case.

SHI, PO are the angles of inclination is contained between two adjacent lines in the coronal plane, situated symmetrically on the left and right body sides; SHI and PO have a value ranging from -180° to 0° when the line of the right side is higher than that of the left or from 0° to 180° when the left side is higher than that of the right.

SHR is the angles contained between two adjacent situated within the coronal plane and a line connecting two points lying on the back surface, situated symmetrically on the left and on the right body sides. SHR and PR values range from 0° to 180° if the right side is rotated far forward and ranges from -180° to 0° in the opposite case.

LL is the angle contained between two adjacent lines as follows: a line is connecting S1 with *L*max and a line connecting *L*max with Kmax. If *L*max is anterior to a line C7-S1, LL value ranges from -180° to 0°; conversely, the LL value ranges from 180° to 0° in the opposite case.

KL index- differences between angles of kyphosis and angle of lordosis. If K is greater than LL, KL index has the positive (+) value, and negative (−) value in the opposite case.

**Table 2:** Description of variable set gait kinematics (criterion set).

Gait kinematics	Mean±SD	Median	Maximum	Minimum
FO (%)	62.16±8.77	61.00	82.00	39.00
MeanPT (°)	9.66±6.38	9.46	22.58	-4.15
MeanPR (°)	4.91±3.19	4.21	14.92	0.03
HMinF (°)	9.03±5.17	8.94	23.51	0.24
HROMF/E (°)	33.91±7.75	33.00	57.75	20.10
HMeanRst (°)	6.42±4.96	5.44	25.90	0.26
HPAbsw (°)	10.59±10.67	6.77	56.58	0.10
KICF (°)	14.05±11.74	12.24	48.80	-6.90
KROMF (°)	50.93±8.33	51.26	72.05	11.70
PDFst (°)	6.13±13.42	11.80	24.96	-24.96
PDFsw (°)	7.87±4.59	7.30	21.10	0.06
FMeanP (°)	11.83±5.87	12.14	29.09	1.45

Gait kinematics (distinct gait parameters that composing GGI): stance phase, expressed as the percentage of the gait cycle (FO); mean of pelvic tilt (MeanPT); mean pelvic rotation (MeanPR); minimum hip flexion (HMinF); ROM of hip flexion/extension (HROMF/E); mean hip rotation in stance (HMeanRst); peak hip abduction in swing (HPAbsw); knee flexion at initial contact (KICF); ROM of knee flexion (KROMF); peak dorsiflexion in stance (PDFst); peak dorsiflexion in swing (PDFsw); mean foot progression (FMeanP)

Results for the structural equation model showed significant *F* (117, 618.88) = 2.21 (*p* < 0.001, λ = 0.07) and yielded nine functions, wherein the *R*^*2*^_*c*_ value was 0.58, 0.48, 0.30, 0.25, 0.16, 0.12, 0.07, 0.03, and 0.01 for each successive function. Results of dimension-reduction analysis showed that functions 1–9 were significant (results for the full model), and functions 2–9 showed significant *F* (96, 562.65) = 1.70, *p* < 0.001. Tests for other functions were not significant at α = 0.05, which indicated that they did not show a significant amount of shared variance between the variable sets. Hence, in further analysis, only Functions 1 and 2 were included. Table 3 presents the standardised canonical function coefficients and structure coefficients for Functions 1 and 2. The squared structure coefficients are also given in addition to the communalities across the Function 1 and 2 for each variable.

**Table 3:** Canonical solution for body posture deviation (MT indices) predicting gait disorders (gait kinematics) for functions 1 and 2.

Variables	Function 1			Function 2		
	Coef.	r_s_	r_s_^2^ (%)	Coef.	r_s_	r_s_^2^ (%)
MT indices						
TI (mm)	0.12	0.03	0.09	-0.44*	-0.40*	16.00
TT (°)	0.06	-0.17	2.89	0.22	0.29	8.41
ACL (°)	-0.13	-0.25	6.25	-0.18	0.08	0.64
PO (°)	-0.09	0.01	0.01	-0.75*	-0.55*	30.25
PR (°)	-0.07	0.11	1.21	0.33	0.02	0.04
SHR (°)	-0.20	-0.12	1.44	0.42*	0.40*	16.00
SHI (°)	-0.17	-0.10	1.00	-0.47*	-0.46*	21.16
LL (°)	0.56*	0.56*	33.36	-0.56*	-0.22	4.84
KL (°)	0.94*	0.86*	73.96	0.16	0.08	0.64
Gait Kinematics						
FO (%)	-0.07	-0.13	1.69	0.20	0.22	4.84
MeanPT (°)	0.43*	0.53*	28.09	-0.19	-0.13	1.69
MeanPR (°)	0.42*	0.44*	19.36	-0.36*	-0.40*	16.00
HMinF (°)	0.39*	0.46*	12.96	-0.23	-0.20	4.00
HROMF/E (°)	0.11	-0.17	2.89	0.01	0.05	0.25
HMeanRst (°)	-0.37	-0.04	0.16	0.43*	0.22	4.84
HPAbsw (°)	-0.14	-0.03	0.09	-0.41*	-0.55*	30.25
KICF (°)	-0.43*	-0.42*	17.64	0.44*	0.35*	12.25
KROMF (°)	-0.10	-0.21	4.41	0.39*	0.56*	31.36
PDFst (°)	-0.79*	-0.85*	72.25	-0.53*	-0.35*	12.25
PDFsw (°)	0.07	-0.17	2.89	-0.02	0.10	1.00
FMeanP (°)	0.09	0.04	0.16	-0.46*	-0.45*	20.25

*p < 0.05.

Coef = standardized canonical function, r_s_ = structure coefficient, r_s_^2^ = squared structure coefficient. The MT indices: trunk inclination index in the sagittal plane (TI); angle of trunk inclination in the sagittal A-P plane (TT); angle of vertebral lateral curvature (ALC); pelvic obliquity (PO); angle of shoulder line inclination in the coronal M-L plane (SHI); angle of shoulder rotation (SHR); angle of pelvic rotation (PR); angle of lordosis (LL); difference between angle of Kyphosis and angle of Lordosis (KL index). Gait kinematics (distinct gait parameters that composed the GGI: FO—stance phase, expressed as the percentage of the gait cycle (FO); mean of pelvic tilt (MeanPT); mean pelvic rotation (MeanPR); minimum hip flexion (HMinF); ROM of hip flexion/extension (HROMF/E); mean hip rotation in stance (HMeanRst); peak hip abduction in swing (HPAbsw); knee flexion at initial contact (KICF); ROM of knee flexion (KROMF); peak dorsiflexion in stance (PDFst); peak dorsiflexion in swing (PDFsw); mean foot progression (FMeanP).

**Fig 1:**
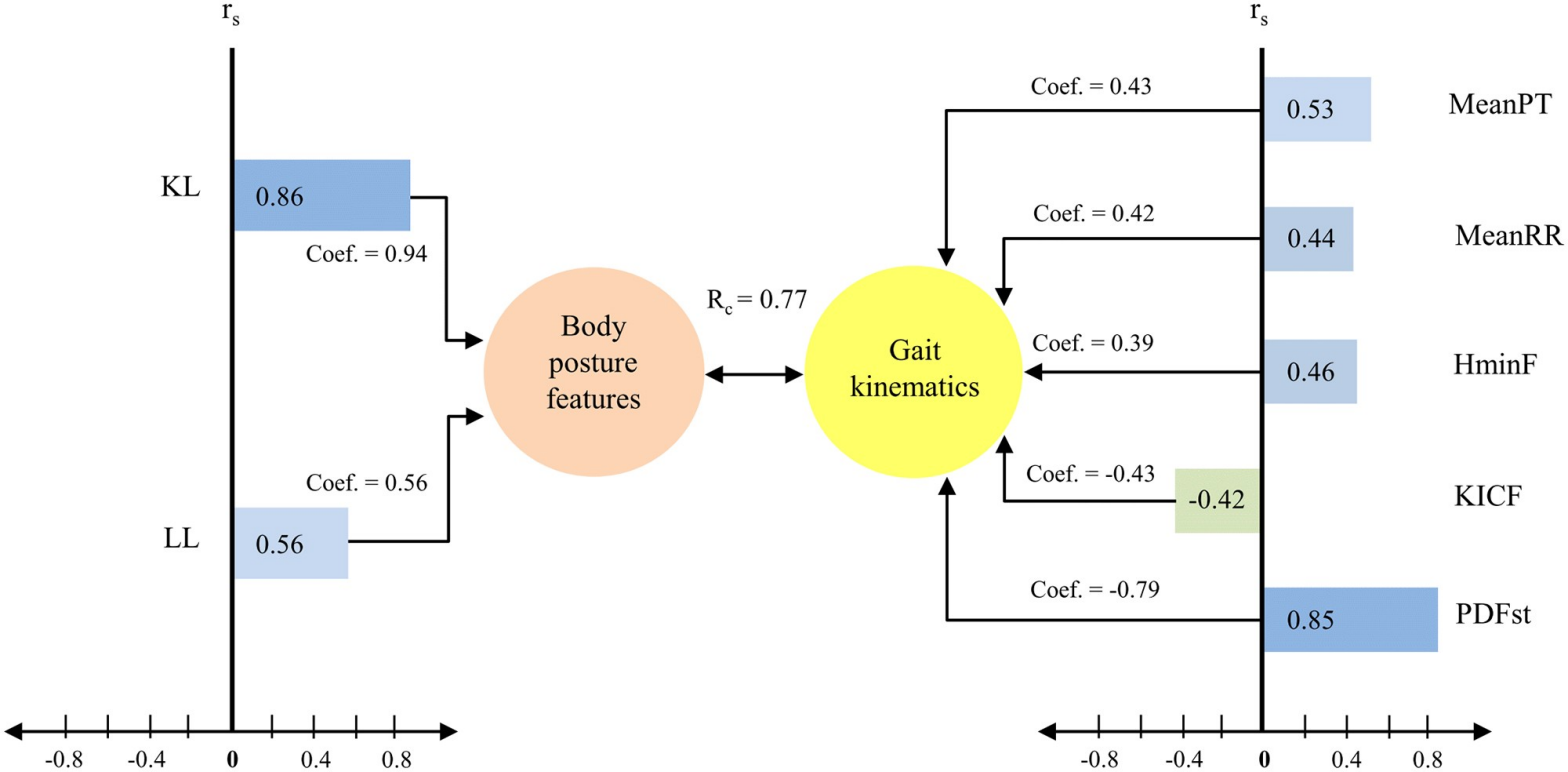
Illustration of the first function in a canonical correlation analysis with a predictor variable set with two variables (body posture features) and criterion variable set with five variables (gait kinematics). Coef = standardized canonical function, r_s_ = structure coefficient, r_s_^2^ = squared structure coefficient. The canonical correlation (R_c_) is a simple Pearson correlation (r) between the synthetic variables, which were linearly combined from the observed variables. KL index, difference between angle of kyphosis and angle of lordosis; LL, angle of lordosis (LL); MeanPT, mean of pelvic tilt; MeanPR, mean pelvic rotation; HMinF, minimum hip flexion; KICF, knee flexion at initial contact; PDFst, peak dorsiflexion in stance.

Looking at the coefficients for Function 1 reveals that relevant criterion variables were mean of pelvic tilt, mean pelvic rotation, minimum hip flexion, knee flexion at initial contact, and peak dorsiflexion in stance, which made secondary contributions to the criterion variable. This conclusion was supported by the squared structure coefficients. The first three variables had the same sign and the last two were inversely related to the criterion set, which also indicated that they were negatively associated with the predictor set (Fig 1).

The other side of the equation on Function 1 involves the predictor set (based on the criterion *r*_*s*_ > 0.40). Table 1 indicates that the difference between angle of kyphosis and angle of lordosis (KL index) and the angle of lordosis variables were the primary contributors to the predictor synthetic variable, with a secondary contribution by gait kinematics. This conclusion was supported by the squared structure coefficients. The structure coefficients of both variables had the same sign, indicating that they were positively associated with the MT indices set (Fig 1).

With regard to the predictor variable set in Function 1, KL index and angle of lordosis variables were positively associated with pelvic and hip kinematics (mean of pelvic tilt, mean pelvic rotation, and minimum hip flexion), and negatively with knee flexion at initial contact and peak dorsiflexion in stance. Taking in to account, that all significant factors of predictors set mainly (except one) referred to the sagittal plane, we labelled Function 1 as ‘postural and gait complex of disorders in the sagittal plane’.

The canonical correlation (R_c_) between both synthetic variables presented a strong uphill (positive) linear relationship.

**Fig 2:**
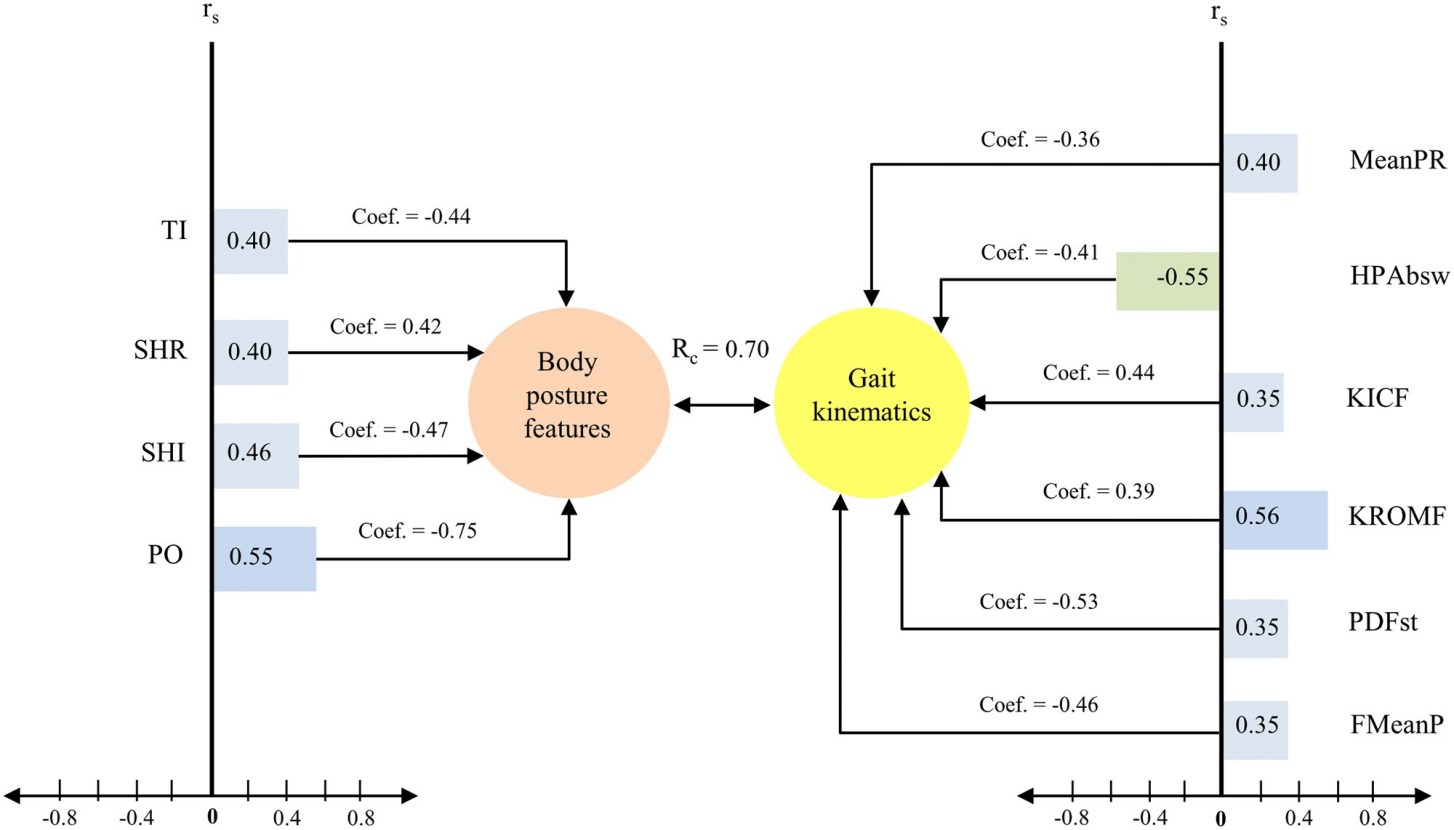
Illustration of the second function in CCA with a predictor variable set with four variables (body posture features) and criterion variable set with six variables (gait kinematics). Coef = standardized canonical function, r_s_ = structure coefficient, r_s_^2^ = squared structure coefficient. The canonical correlation (R_c_) is a simple Pearson correlation (r) between the synthetic variables, which were linearly combined from the observed variables. TI, trunk inclination index in the coronal plane; SHR, angle of shoulder rotation; SHI, angle of shoulder line inclination in the coronal plane; PO, pelvic obliquity; MeanPR, mean pelvic rotation; HPAbsw, peak hip abduction in swing; KICF, knee flexion at initial contact; KROMF, ROM of knee flexion; PDFst, peak dorsiflexion in stance; FMeanP, mean foot progression.

For Function 2, the coefficients in Table 3 suggest that the only criterion variables of relevance were mean pelvic rotation, peak hip abduction in swing, peak dorsiflexion in stance, mean foot progression, knee flexion at initial contact, and ROM of knee flexion, albeit less for the latter. Knee flexion at initial contact and ROM of knee flexion were characterised by a positive sign and were inversely associated with the other variables (Fig 2). Regarding the predictor variable set in Function 2, the variables: trunk inclination index in the coronal plane, pelvic obliquity, angle of shoulder rotation, and angle of shoulder line inclination in the coronal plane were the primary contributors to the predictor synthetic variable, with secondary contribution by gait kinematics. In the predictor set, only angle of shoulder rotation showed a positive sign and was inversely associated with other predictors (Table 3). The postural features such as trunk inclination index in the coronal plane, pelvic obliquity, and angle of shoulder inclination and angle of shoulder rotation were positively associated with pelvic and hip kinematics (mean of pelvic tilt, mean pelvic rotation, and minimum hip flexion), but negatively associated with knee flexion at initial contact and peak dorsiflexion in stance (Fig 2). Because significant factors of predictors set mainly (except one) referred to the coronal plane, therefore, this function seem to capture theoretically consistent relationships that one may collectively call ‘postural and gait complex of disorders in the coronal plane’.

## 4. Discussion

Although some of our previous studies have shown that body-posture deviations and gait disorders in children with CP are related, the relationship between them was exploring separately in clinical subgroups (UCP/BCP) [13–16]. Taken together, these findings revealed that abnormal postural patterns can be a major component of gait disorders in children with UCP [13,14] and BCP [15,16].

Although this was not the main focus of this research, factorial analysis used to reduce the number of variables of gait parameters identified previously unreported kinematic deviations in these children, including pelvic misalignment in all (sagittal, frontal, and horizontal) planes and an inadequate ROM of hip abduction/adduction in swing phase. Scholars have highlighted that gait problems in children with UCP and BCP mainly involve deviations in lower-limb gait kinematics in the sagittal plane, without accounting for pelvic kinematics and truncal posture [28–31].

Although surface topography and quantitative 3DIGA are excellent indicators of postural dysfunction and gait disorders in patients with CP, respectively, both methods generate a considerable amount of data that require complex interpretation. To overcome these problems, we used CCA to jointly analyse multiple datasets to establish a multivariate relationship between MT indices (predictor set) and gait kinematics (criterion set). Exploration of the relationship between body-posture deviation and gait disturbances in children with CP *via* CCA showed that the most important postural feature related to gait disturbances in this population was sagittal misalignment of the spine. That is, inadequate curvature (kyphosis or lordosis) construction of the spine expressed by the magnitude of angle of lordosis and KL index. When the angle of kyphosis is larger than lordosis, the KL index ranges from −180° to 0° and, conversely, ranges from 180° to 0° in the opposite case.

Our findings documented that sagittal misalignment of the spine curvature was related significantly to kinematic deviations during the gait cycle, such as deviations of pelvic, hip, knee, and ankle kinematics (express by mean pelvic tilt, inadequate hip abduction in swing phase, knee flexion at initial contact, and peak dorsiflexion in stance, respectively), which mainly suggests association with gait disturbances in the sagittal plane. Hyperlordosis tended to involve not only abnormalities of pelvic and hip movements during gait (theoretically expected relationships) but also abnormalities of the knee (knee flexion at initial contact) and ankle (peak dorsiflexion in stance) movements. Moreover, the obtained results showed not only well-documented key interactions between adjacent segments of lower limbs during gait, but also the relevant opposite interrelationship between pelvic and hip movements with distant segment kinematics (e.g., knee flexion and ankle dorsiflexion in stance). Given that the features of the sagittal plane, both in the predictor and criterion variables, were the dominant contributors, one can label this relationship collectively as ‘postural and gait complex of disorders in the sagittal plane’.

Function 2 also yielded theoretically expected relationships between deviations in body posture and gait disorders in children with CP, as expressed by the strong positive linear relationship between both synthetic variables. We also drew knowledge from the predictor variables used in the second model. These obtained results showed that the static misalignment of particular segments of body posture in the coronal plane expressed by the trunk inclination index, angle of shoulder line inclination, and pelvic obliquity were the primary contributors to the predictor synthetic variables, with a secondary contribution by development of gait disturbances in children with CP. Our findings documented that coronal misalignment of the pelvis, trunk and shoulder girdle was significantly and positively associated with kinematic deviations during the gait cycle, such as mean pelvic rotation, peak hip abduction in swing, knee flexion at initial contact, ROM of knee flexion, peak dorsiflexion in stance, and mean foot progression. Because the postural predictors in the coronal plane were the dominant contributors, we can label this relationship collectively as ‘postural and gait complex of disorders in the coronal plane’.

The results of the present study are supportive of data from our previous study exploring an association between posture and gait separately in clinical subgroups (UCP/BCP) [13–16]. Those studies documented that the discrepancy in gait among children with UCP and children with BCP was not simply a lower-limb kinematic deviation in the sagittal plane [28–31]. The gait patterns were also dependent upon kinematic deviations resulting directly from two postural-pattern features. The first was static misalignments of the trunk and pelvis in the sagittal plane (e.g. those resulting from excessive anterior pelvic tilt and excessive lumbar curve in the spine) of children with BCP [15]. The second was static misalignments of the trunk, shoulder girdle, and pelvis in the coronal plane (asymmetry) (e.g., spine inclination towards the unaffected/affected side, pelvis obliquity down/up on the affected side, and shoulder inclination towards the unaffected/affected side) in children with UCP.

Although the obtained results revealed the significant multivariate shared relationship between specific postural deviations and specified gait disturbances in children with CP, the causal relationships between them is not known. The clinical applications of this study relate to the early recognition of particular features of postural deviation using surface topography, instead of more difficult and demanding expensive tools 3-D gait analysis.

Attention should be paid to promote and facilitate the development of: (i) appropriate curvature (kyphosis or lordosis) construction in the spine and neutral pelvis position in the sagittal plane, and the symmetry of pelvic, trunk, and shoulder girdle orientation in the coronal plane; (ii) motor control of pelvic rotation, hip abduction, knee flexion, and ankle dorsiflexion.

Our study had three main limitations. First, the ability to walk without assistive devices was a criterion for study inclusion. Second, previous lower-limb surgery was an exclusion criterion, which is why only some children with CP were included. Third, our study was based on an incomplete set of the 16 gait parameters that comprise the GGI [32]. The complete analysis should involve all the spatiotemporal variables and kinematic parameters from 3DIGA, but so much data is generated that such complex interpretation is not possible in this type of study. To overcome these problems, a set of 16 gait parameters closely correlated with particular gait problems in children with CP were selected by a group of experienced clinicians and which are included in the present study [24].

## 5. Conclusions

Based on present results and referring to our previous finding documents that deviation from the proper posture in the children with BCP mostly included postural features characteristic of the sagittal plane [15] whereas, the asymmetric alignment of segments of body posture in a coronal plane is mostly characteristic of children with a UCP [13], it can be the assumed that:

1. the first model of relationship between postural deviation and gait disturbances, termed ‘postural and gait complex of disorders in the sagittal plane’, was related to children with BCP,
2. the second model, ‘postural and gait complex of disorders in the coronal plane’, illustrated these relationships in children with UCP.

Although, the results of the current study seem promising, the relationships between gait & posture should be further explored in different subgroups of children with CP.

## Supporting information

S1 Data (XLSX)

S1 File (PDF)
