# Supplementary material for: Canonical correlation between body-posture deviations and gait disorders in children with cerebral palsy
Source: PLoS One. 2020 Jun 16;15(6):e0234654. doi: 10.1371/journal.pone.0234654 (PMC7297316; doi:10.1371/journal.pone.0234654)
Supplement: S1 File — (PDF) [file pone.0234654.s002.pdf]

```
 * Encoding: UTF-8.
MANOVA Ch_FO_ZL Ch_MPT_ZL A_Ch_MPR_HP A_Ch_MHF_HP Ch_HRFE_ZL A_Ch_StMHR_ZL
A_Ch_StMHR_HP Ch_ICKFL_ZL Ch_RkFlEx_HP Ch_PDST_HP A_Ch_PDSW_HP A_Ch_P_HP Ch
_SL_HP WITH DCK A_KPT KNT A_KNT KNM KSB A_KSB LL_Kat MI
/DISCRIM all alpha(1)
/print=SIGNIF(eigen dim).
```

Manova

Notes

Output Created		12-OCT-2018 14:19:01
Comments		
Input	Data	D:\Praca\PogotowieStatystyczne\Klienci\Andrzej Szopa\Dane20150923-bez skolioz.sav
	Active Dataset	DataSet1
	Filter	DIAG ~= 104 (FILTER)
	Weight	<none>
	Split File	<none>
	N of Rows in Working Data File	120
Syntax		MANOVA Ch_FO_ZL Ch_MPT_ZL A_Ch_MPR_HP A_Ch_MHF_HP Ch_HRFE_ZL A_Ch_StMHR_ZL A_Ch_StMHR_HP Ch_ICKFL_ZL Ch_RkFlEx_HP Ch_PDST_HP A_Ch_PDSW_HP A_Ch_P_HP Ch_SL_HP WITH DCK A_KPT KNT A_KNT KNM KSB A_KSB LL_Kat MI /DISCRIM all alpha(1) /print=SIGNIF(eigen dim).
Resources	Processor Time	00:00:00,02
	Elapsed Time	00:00:00,02

```
 - - - - - - - - - - - - - - - - - - - - - - - - - - - - - - - - - - - - -
- - - - - - - - - - - - - - - - - - - - - - - -
The default error term in MANOVA has been changed from WITHIN CELLS to
WITHIN+RESIDUAL.  Note that these are the same for all full factorial desig
ns.
```

* * * * * * * * * * * * * * * * * * A n a l y s i s   o f   V a r i a n c e * * * * * * * * * * * * * * * * * *

```
      120 cases accepted.
        0 cases rejected because of out-of-range factor values.
        0 cases rejected because of missing data.
        1 non-empty cell.

        1 design will be processed.
```

- -

* * * * * * * * * * * * * * * * * * A n a l y s i s   o f   V a r i a n c e -- Design 1 * * * * * * * * * * * * * * * * * *

EFFECT .. WITHIN CELLS Regression
Multivariate Tests of Significance (S = 9, M = 1 1/2, N = 39 1/2)

Test Name	Value	Approx. F	Hypoth. DF	Error DF	Sig. of F
Pillais	2,00875	1,96706	117,00	801,00	,000
Hotellings	3,58484	2,42734	117,00	713,00	,000
Wilks	,07355	2,21065	117,00	618,88	,000
Roys	,59175				

- -

Eigenvalues and Canonical Correlations

Root No.	Eigenvalue	Pct.	Cum. Pct.	Canon Cor.	Sq. Cor
1	1,44950	40,43424	40,43424	,76926	,59175
2	,93752	26,15231	66,58655	,69561	,48388
3	,43371	12,09850	78,68504	,55001	,30251
4	,32684	9,11731	87,80236	,49632	,24633
5	,19478	5,43331	93,23567	,40376	,16302
6	,13174	3,67493	96,91059	,34118	,11641
7	,07210	2,01129	98,92189	,25933	,06725
8	,03233	,90174	99,82362	,17696	,03131
9	,00632	,17638	100,00000	,07927	,00628

- -

Dimension Reduction Analysis

Roots	Wilks L.	F	Hypoth. DF	Error DF	Sig. of F
1 TO 9	,07355	2,21065	117,00	618,88	,000
2 TO 9	,18016	1,69803	96,00	562,65	,000
3 TO 9	,34906	1,25883	77,00	504,81	,079
4 TO 9	,50045	1,04800	60,00	445,16	,385
5 TO 9	,66401	,81652	45,00	383,33	,795
6 TO 9	,79335	,64531	32,00	318,75	,933
7 TO 9	,89786	,45582	21,00	250,37	,982
8 TO 9	,96260	,28221	12,00	176,00	,991
9 TO 9	,99372	,11255	5,00	89,00	,989

- -

EFFECT .. WITHIN CELLS Regression (Cont.)
Univariate F-tests with (9;93) D. F.

Variable	Sq. Mul. R	Adj. R-sq.	Hypoth. MS	Error MS	F	Sig. of F
Ch_FO_ZL	,13365	,04981	115,69939	72,58072	1,59408	,128
Ch_MPT_Z	,22127	,14591	68,25944	23,24794	2,93615	,004
A_Ch_MPR	,19978	,12234	29,11528	11,28570	2,57984	,011
A_Ch_MHF	,15787	,07637	86,36810	44,58677	1,93708	,056
Ch_HRFE_	,06244	,00000	48,45874	70,41317	,68821	,718

```
 A_Ch_StM           ,06489          ,00000       20,10137        28,03453
     ,71702          ,692
 A_Ch_S_1           ,19455          ,11660      257,09417       103,00605
    2,49591          ,013
 Ch_ICKFL           ,29208          ,22357      342,57938        80,35506
    4,26332          ,000
 Ch_RkFlE           ,23521          ,16120      222,50042        70,01300
    3,17799          ,002
 Ch_PDST_           ,50536          ,45749     1139,81599       107,96594
   10,55718          ,000
 A_Ch_PDS           ,06476          ,00000       19,29631        26,96961
     ,71548          ,693
 A_Ch_P_H           ,09297          ,00519       50,90380        48,06239
    1,05912          ,400
 Ch_SL_HP           ,08435          ,00000         ,00563          ,00591
     ,95189          ,485

 - - - - - - - - - - - - - - - - - - - - - - - - - - - - - - - - - - - - -
- - - - - - - - - - - - - - - - - - - - - - -
 Raw canonical coefficients for DEPENDENT variables
           Function No.

 Variable                 1                2                3
   4                5                6                7

 Ch_FO_ZL             -,00827           ,02303          -,05126           ,0
6584          -,04046          -,00889          -,00203
 Ch_MPT_Z              ,04441          -,03657          -,03096           ,0
3664          -,15740           ,08365           ,05031
 A_Ch_MPR              ,03275          -,10073           ,00926           ,1
7749           ,14885           ,03611          -,11399
 A_Ch_MHF              ,01145          -,03301          -,01429           ,0
5669           ,02529           ,00499           ,09345
 Ch_HRFE_              ,01309           ,00112           ,01624           ,0
1319           ,03327          -,01545           ,08407
 A_Ch_StM             -,07087           ,08257          -,04714          -,0
1602           ,02871          -,03780           ,07825
 A_Ch_S_1             -,01257          -,02891           ,03083           ,0
0717           ,00287           ,01391           ,00139
 Ch_ICKFL             -,02274           ,00352           ,08748           ,0
3638          -,01121           ,02610           ,02256
 Ch_RkFlE             -,01125           ,04226          -,03668           ,0
2291           ,02625           ,06116          -,03545
 Ch_PDST_             -,05585          -,03766          -,02392           ,0
2035          -,02042           ,00724          -,00037
```

A_Ch_PDS ,01396 -,00451 ,00479 -,0
5672 ,04950 -,05738 ,04001
A_Ch_P_H ,01332 -,03793 -,02305 -,0
4294 ,03428 ,05004 ,00684
Ch_SL_HP 3,90675 -,87633 ,84304 ,5
4125 -,04674 -8,08350 -5,56473

Variable	8	9
Ch_FO_ZL	-,00928	-,02809
Ch_MPT_Z	,06373	,00619
A_Ch_MPR	,03346	-,03472
A_Ch_MHF	,06691	-,01278
Ch_HRFE_	-,06216	-,01275
A_Ch_StM	,03877	,06932
A_Ch_S_1	-,03358	-,04759
Ch_ICKFL	,04081	,02819
Ch_RkFlE	-,02047	-,04685
Ch_PDST_	,02661	,01412
A_Ch_PDS	,08069	-,15423
A_Ch_P_H	-,00694	,02374
Ch_SL_HP	-,20775	2,24452

\- -
\- -

Standardized canonical coefficients for DEPENDENT variables
Function No.

Variable 1 2 3
4 5 6 7

Ch_FO_ZL -,07224 ,20130 -,44799 ,5
7547 -,35359 -,07771 -,01774
Ch_MPT_Z ,23169 -,19081 -,16153 ,1
9116 -,82120 ,43642 ,26250
A_Ch_MPR ,11743 -,36121 ,03320 ,6
3647 ,53378 ,12948 -,40876
A_Ch_MHF ,07952 -,22936 -,09927 ,3
9385 ,17571 ,03470 ,64927
Ch_HRFE_ ,10832 ,00930 ,13441 ,1
0918 ,27529 -,12788 ,69564
A_Ch_StM -,37050 ,43170 -,24646 -,0
8374 ,15009 -,19761 ,40910
A_Ch_S_1 -,13576 -,31213 ,33286 ,0
7747 ,03099 ,15025 ,01504

Ch_ICKFL	-,23136	,03582	,88991	,37006	-,11399	,26554	,22951
Ch_RkFlE	-,10280	,38608	-,33515	,20928	,23986	,55880	-,32384
Ch_PDST_	-,78784	-,53128	-,33746	,28713	-,28808	,10208	-,00528
A_Ch_PDS	,07158	-,02310	,02459	-,29083	,25380	-,29420	,20513
A_Ch_P_H	,09256	-,26364	-,16020	-,29849	,23828	,34785	,04753
Ch_SL_HP	,29970	-,06723	,06467	,04152	-,00359	-,62012	-,42689

Variable	8	9
Ch_FO_ZL	-,08112	-,24553
Ch_MPT_Z	,33248	,03232
A_Ch_MPR	,11997	-,12451
A_Ch_MHF	,46488	-,08877
Ch_HRFE_	-,51436	-,10555
A_Ch_StM	,20271	,36244
A_Ch_S_1	-,36264	-,51385
Ch_ICKFL	,41516	,28682
Ch_RkFlE	-,18697	-,42802
Ch_PDST_	,37546	,19921
A_Ch_PDS	,41376	-,79084
A_Ch_P_H	-,04824	,16499
Ch_SL_HP	-,01594	,17219

- -

Correlations between DEPENDENT and canonical variables

Function No.

Variable	1	2	3	4	5	6	7
Ch_FO_ZL	-,13491	,22310	-,05975	,51130	-,37251	-,28153	-,09483
Ch_MPT_Z	,52522	-,12512	-,10309	,11386	-,41118	,30574	,25862
A_Ch_MPR	,33759	-,29676	-,00083	,45950	,46109	,09499	-,11212
A_Ch_MHF	,35977	-,19977	-,28239	,27448	,24991	,00605	,31221

```
 Ch_HRFE_         -,17381        ,05005        -,00445         ,1
6113         ,12229        -,14030        ,55543
 A_Ch_StM         -,03666        ,22523        -,09590         ,2
0735         ,30890        -,09821        ,32188
 A_Ch_S_1         -,03185       -,54924         ,32561         ,2
1126        -,06695         ,01741        ,07114
 Ch_ICKFL         -,31771        ,35415         ,71253         ,2
3759        -,05396         ,12269       -,05800
 Ch_RkFlE         -,21306        ,56063        -,22089         ,1
5398         ,24772         ,44479       -,18096
 Ch_PDST_         -,84773       -,35310        -,23187        -,0
3745        -,07902        -,13037       -,04366
 A_Ch_PDS         -,17403        ,10138         ,10716        -,2
9319         ,05368        -,23237        ,05206
 A_Ch_P_H          ,04169       -,34684        -,04155        -,1
9400         ,25510         ,33298       -,03984
 Ch_SL_HP          ,15533       -,04827         ,02309         ,3
4386         ,03243        -,54861       -,25405

 Variable               8             9

 Ch_FO_ZL         -,13084       -,22885
 Ch_MPT_Z          ,18764        ,02847
 A_Ch_MPR          ,18319        ,13428
 A_Ch_MHF          ,28476        ,01773
 Ch_HRFE_         -,60529       -,01541
 A_Ch_StM          ,23971        ,34038
 A_Ch_S_1         -,32450       -,28558
 Ch_ICKFL          ,23331        ,03738
 Ch_RkFlE         -,04069       -,24446
 Ch_PDST_         -,03201       -,02478
 A_Ch_PDS          ,49134       -,65744
 A_Ch_P_H          ,01327        ,22142
 Ch_SL_HP          ,00475        ,13102

 - - - - - - - - - - - - - - - - - - - - - - - - - - - - - - - - - - - - -
- - - - - - - - - - - - - - - - - - - - - - -
 Variance in dependent variables explained by canonical variables

 CAN. VAR.    Pct Var DEP    Cum Pct DEP    Pct Var COV    Cum Pct
 COV

       1       11,47034       11,47034        6,78762        6,7
8762
```

2	9,58162	21,05196	4,63632	11,42393
3	6,40910	27,46106	1,93881	13,36275
4	7,67570	35,13676	1,89076	15,25350
5	6,44065	41,57741	1,04997	16,30348
6	6,97820	48,55560	,81230	17,11577
7	5,46114	54,01675	,36728	17,48305
8	7,65262	61,66937	,23963	17,72268
9	6,37981	68,04918	,04008	17,76277

- -

Raw canonical coefficients for COVARIATES
Function No.

COVARIATE	1	2	3	4	5	6	7
DCK	,00216	-,00433	,00359	-,00778	,00772	-,00788	-,00339
A_KPT	,01271	,04976	,06221	,02607	-,08275	-,12100	,17232
KNT	-,02502	-,03531	,03953	-,08145	-,03280	-,06748	-,06466
A_KNT	-,02626	-,20996	-,10191	-,02952	-,02159	-,00744	,17875
KNM	-,00989	,04661	-,05712	,04278	,04100	-,10141	-,05625
KSB	-,01862	,01979	-,01736	,01612	,07435	,02601	,03714
A_KSB	-,02689	-,07429	-,02021	,11148	,01215	,03497	,00239
LL_Kat	,06078	-,09152	,13035	-,01196	,02532	,00603	,05181
MI	,05619	,00963	-,01247	,00102	-,00671	-,00205	,00219

COVARIATE 8 9

DCK	,01125	,00172
A_KPT	,01904	-,01954
KNT	-,11078	-,20019
A_KNT	-,13656	,00155
KNM	-,07837	-,04296
KSB	-,01299	-,04305
A_KSB	,03036	-,10153
LL_Kat	-,11139	-,05134
MI	,01164	-,01721

- -

Standardized canonical coefficients for COVARIATES
CAN. VAR.

COVARIATE	1	2	3	4	5	6	7
DCK	,12103	-,24312	,20159	-,43608	,43311	-,44167	-,18989
A_KPT	,05506	,21550	,26940	,11290	-,35837	-,52405	,74629
KNT	-,12575	-,17744	,19864	-,40930	-,16483	-,33909	-,32490
A_KNT	-,09366	-,74874	-,36343	-,10526	-,07701	-,02653	,63746
KNM	-,07071	,33329	-,40850	,30593	,29320	-,72519	-,40224
KSB	-,20414	,21696	-,19028	,17675	,81507	,28516	,40715
A_KSB	-,17179	-,47462	-,12913	,71225	,07761	,22340	,01529
LL_Kat	,36875	-,55522	,79083	-,07259	,15361	,03656	,31434
MI	,93830	,16087	-,20817	,01709	-,11209	-,03429	,03660

COVARIATE	8	9
DCK	,63115	,09636
A_KPT	,08245	-,08464
KNT	-,55664	-1,00593
A_KNT	-,48699	,00552
KNM	-,56042	-,30719

KSB	-,14236	-,47195
A_KSB	,19399	-,64865
LL_Kat	-,67577	-,31145
MI	,19439	-,28743

- -

Correlations between COVARIATES and canonical variables

CAN. VAR.

Covariate	1	2	3	4	5	6	7
DCK	,02996	-,40302	,06699	-,26504	,48913	-,51193	-,04887
A_KPT	-,16749	,29167	,26423	,16608	-,28195	-,49692	,61950
KNT	-,25186	,08174	-,04576	-,60268	-,31984	,04245	-,09732
A_KNT	,00553	-,54860	-,65019	-,28667	-,03744	-,13252	,35846
KNM	,11791	,01695	-,16368	,47760	,28884	-,62445	-,26040
KSB	-,12123	,39679	-,11448	-,24649	,61344	,29212	,43574
A_KSB	-,10390	-,45703	,05250	,71662	-,08939	-,03692	-,13338
LL_Kat	,35684	-,21839	,66543	,31045	,29792	-,00403	-,04478
MI	,86177	,08082	-,38432	-,09338	-,07137	,09898	,06104

Covariate	8	9
DCK	,50792	,01556
A_KPT	,23144	-,16035
KNT	,00044	-,67157
A_KNT	-,18016	,11905
KNM	-,37990	,21312
KSB	,00579	-,32008
A_KSB	,36651	-,32024
LL_Kat	-,39992	,18731
MI	,09988	-,25654

- -

Variance in covariates explained by canonical variables

CAN. VAR.	Pct Var DEP	Cum Pct DEP	Pct Var COV	Cum Pct COV
1	6,58679	6,58679	11,13096	11,13096
2	5,24722	11,83401	10,84414	21,97510
3	3,80586	15,63987	12,58095	34,55605
4	3,97066	19,61053	16,11926	50,67531
5	1,78241	21,39294	10,93354	61,60885
6	1,31256	22,70550	11,27581	72,88466
7	,60178	23,30729	8,94806	81,83272
8	,27577	23,58305	8,80671	90,63942
9	,05881	23,64187	9,36058	100,00000

- -

Regression analysis for WITHIN CELLS error term
--- Individual Univariate ,9500 confidence intervals
Dependent variable .. Ch_FO_ZL Ch_FO_ZL: foot off % kd hemi zdrowa/lewa

COVARIATE	B	Beta	Std. Err.	t-Value	Sig. of t	Lower -95%	CL- Upper
DCK	-,0314761521	-,2019866847	,01599	-1,96790	,052	-,06324	,00029
A_KPT	,2684644905	,1330334168	,20509	1,30899	,194	-,13881	,67574
KNT	-,0493850207	-,0283930666	,22671	-,21783	,828	-,49959	,40082
A_KNT	-,2735467521	-,1116146999	,27613	-,99063	,324	-,82189	,27480

KNM	,2494688999	,2041188015	,14727	1,69396	,094	-,04298	,54192
KSB	-,0336247522	-,0421754516	,08809	-,38169	,704	-,20856	,14131
A_KSB	,1408021915	,1029269204	,15015	,93772	,351	-,15737	,43898
LL_Kat	-,2620310848	-,1818925945	,18406	-1,42365	,158	-,62753	,10347
MI	-,0215966975	-,0412635239	,05263	-,41036	,682	-,12611	,08291

Dependent variable .. Ch_MPT_ZL   Ch_MPT_ZL: mean pelvis tilt kd he mi zdro

COVARIATE	B	Beta	Std. Err.	t-Value	Sig. of t	Lower -95%	CL- Upper
DCK	-,0070280643	-,0755511921	,00905	-,77638	,439	-,02500	,01095
A_KPT	,0626720968	,0520250626	,11607	,53993	,591	-,16783	,29317
KNT	-,1249100028	-,1203036406	,12831	-,97352	,333	-,37970	,12988
A_KNT	,1149872531	,0785966479	,15628	,73578	,464	-,19535	,42533
KNM	-,1369622807	-,1877293033	,08335	-1,64326	,104	-,30247	,02855
KSB	-,0783557735	-,1646402976	,04986	-1,57161	,119	-,17736	,02065
A_KSB	,0293094527	,0358915129	,08498	,34490	,731	-,13944	,19806
LL_Kat	,1071646539	,1246172777	,10417	1,02877	,306	-,09969	,31402
MI	,1253353935	,4011595994	,02979	4,20795	,000	,06619	,18448

Dependent variable .. A_Ch_MPR_HP   A_Ch_MPR_HP: =Abs(Ch_MPR_HP)

COVARIATE	B	Beta	Std. Err.	t-Value	Sig. of t	Lower -95%	CL- Upper
DCK	,0048210548	,0754025691	,00631	,76438	,447	-,00770	,01735
A_KPT	-,0895810638	-,1081914853	,08087	-1,10767	,271	-,25018	,07102
KNT	-,1073606912	-,1504406952	,08940	-1,20094	,233	-,28489	,07016

```
 A_KNT          ,0572602435    ,0569437411          ,10889          ,52587
       ,600          -,15897          ,27349
 KNM            ,0020712573    ,0041305095          ,05807          ,03567
       ,972          -,11325          ,11739
 KSB            ,0268568156    ,0821028320          ,03474          ,77314
       ,441          -,04212          ,09584
 A_KSB          ,1327327132    ,2364833172          ,05921         2,24176
       ,027           ,01515          ,25031
 LL_Kat         ,1116412837    ,1888817494          ,07258         1,53823
       ,127          -,03248          ,25577
 MI             ,0418002285    ,1946527738          ,02075         2,01420
       ,047           ,00059          ,08301
 Dependent variable .. A_Ch_MHF_HP          A_Ch_MHF_HP: =Abs(Ch_MHF_HP)

 COVARIATE                   B            Beta      Std. Err.         t-Value
  Sig. of t      Lower -95%      CL- Upper

 DCK            ,0044500563    ,0359217725          ,01254          ,35497
       ,723          -,02044          ,02934
 A_KPT         -,0223854803   -,0139537666          ,16075         -,13926
       ,890          -,34160          ,29683
 KNT           -,2348574259   -,1698527173          ,17769        -1,32173
       ,190          -,58771          ,11800
 A_KNT          ,2717662826    ,1394879042          ,21643         1,25570
       ,212          -,15801          ,70155
 KNM            ,0059147283    ,0060876901          ,11543          ,05124
       ,959          -,22330          ,23513
 KSB            ,0475006698    ,0749465290          ,06905          ,68796
       ,493          -,08961          ,18461
 A_KSB          ,1673543534    ,1538890508          ,11769         1,42203
       ,158          -,06635          ,40106
 LL_Kat         ,0607247731    ,0530248252          ,14426          ,42094
       ,675          -,22575          ,34719
 MI             ,1135695174    ,2729555259          ,04125         2,75326
       ,007           ,03166          ,19548
 Dependent variable .. Ch_HRFE_ZL          Ch_HRFE_ZL: hip ROM F-E   kd hem
i zdrowa

 COVARIATE                   B            Beta      Std. Err.         t-Value
  Sig. of t      Lower -95%      CL- Upper

 DCK           -,0166072474   -,1125574974          ,01575        -1,05415
       ,295          -,04789          ,01468
 A_KPT          ,2190951081    ,1146680013          ,20201         1,08459
       ,281          -,18205          ,62024
```

```
 KNT           -,0007214639   -,0004380941          ,22330          -,00323
       ,997          -,44415          ,44271
 A_KNT          ,2793137936    ,1203698954          ,27198          1,02697
       ,307          -,26078          ,81941
 KNM            ,1136097426    ,0981788273          ,14505           ,78322
       ,435          -,17444          ,40166
 KSB            ,1136173511    ,1505154132          ,08677          1,30943
       ,194          -,05869          ,28592
 A_KSB          ,0506110567    ,0390751484          ,14789           ,34221
       ,733          -,24308          ,34430
 LL_Kat         ,0647840463    ,0474969737          ,18129           ,35736
       ,722          -,29522          ,42478
 MI            -,0679248595   -,1370703063          ,05184         -1,31036
       ,193          -,17086          ,03501
 Dependent variable .. A_Ch_StMHR_ZL          A_Ch_StMHR_ZL: =Abs(Ch_StMHR_
ZL)

 COVARIATE                B           Beta      Std. Err.         t-Value
  Sig. of t     Lower -95%     CL- Upper

 DCK           -,0013679818   -,0146747403          ,00994          -,13762
       ,891          -,02111          ,01837
 A_KPT          ,0796625707    ,0659898615          ,12746           ,62498
       ,534          -,17346          ,33278
 KNT           -,1705571600   -,1639215383          ,14090         -1,21050
       ,229          -,45035          ,10924
 A_KNT         -,1207192300   -,0823408403          ,17161          -,70343
       ,484          -,46151          ,22007
 KNM            ,0749178858    ,1024710334          ,09153           ,81853
       ,415          -,10684          ,25667
 KSB            ,0836003705    ,1752902886          ,05475          1,52696
       ,130          -,02512          ,19232
 A_KSB          ,0039216788    ,0047922624          ,09332           ,04202
       ,967          -,18139          ,18924
 LL_Kat        -,1201861212   -,1394650949          ,11439         -1,05067
       ,296          -,34734          ,10697
 MI             ,0006888185    ,0022000510          ,03271           ,02106
       ,983          -,06426          ,06564
 Dependent variable .. A_Ch_StMHR_HP          A_Ch_StMHR_HP: =Abs(Ch_StMHR_
HP)

 COVARIATE                B           Beta      Std. Err.         t-Value
  Sig. of t     Lower -95%     CL- Upper
```

DCK	,0046292812	,0240439857	,01905	,24295	,809	-,03321	,04247
A_KPT	-,0151449288	-,0060742537	,24433	-,06199	,951	-,50033	,47004
KNT	,2465202643	,1147153354	,27008	,91277	,364	-,28980	,78284
A_KNT	,7683842711	,2537582050	,32896	2,33582	,022	,11514	1,42163
KNM	-,2222801436	-,1472038134	,17544	-1,26697	,208	-,57067	,12611
KSB	-,0860940516	-,0874028490	,10495	-,82037	,414	-,29450	,12231
A_KSB	,4058889253	,2401477712	,17888	2,26908	,026	,05067	,76110
LL_Kat	,6849335942	,3848242793	,21927	3,12375	,002	,24951	1,12035
MI	-,0783059332	-,1210947826	,06270	-1,24897	,215	-,20281	,04620

Dependent variable .. Ch_ICKFL_ZL   Ch_ICKFL_ZL: IC knee FL kd hemi zdrowa/

COVARIATE	B	Beta	Std. Err.	t-Value	Sig. of t	Lower -95%	CL- Upper
DCK	-,0109951722	-,0606168831	,01683	-,65332	,515	-,04442	,02243
A_KPT	,3202190183	,1363237354	,21580	1,48388	,141	-,10831	,74875
KNT	-,0305207886	-,0150752066	,23854	-,12795	,898	-,50422	,44318
A_KNT	-,9856447548	-,3455100695	,29055	-3,39239	,001	-1,56261	-,40868
KNM	-,1129114440	-,0793697514	,15496	-,72867	,468	-,42062	,19480
KSB	,0282013159	,0303892851	,09269	,30425	,762	-,15587	,21227
A_KSB	-,0446457872	-,0280382246	,15799	-,28258	,778	-,35838	,26909
LL_Kat	,0655640012	,0391001220	,19366	,33855	,736	-,31901	,45014
MI	-,1593847251	-,2616231238	,05538	-2,87825	,005	-,26935	-,04942

Dependent variable .. Ch_RkFlEx_HP   Ch_RkFlEx_HP: ROM knee Fl-EX hemi/prawa

COVARIATE	B	Beta	Std. Err.	t-Value	Sig. of t	Lower -95%	CL- Upper
DCK	-,0315490491	-,1936746941	,01571	-2,00830	,048	-,06274	-,00035
A_KPT	-,2076417331	-,0984314854	,20143	-1,03082	,305	-,60765	,19236
KNT	-,2421826618	-,1332004240	,22266	-1,08766	,280	-,68435	,19998
A_KNT	-,7141987928	-,2787753974	,27120	-2,63343	,010	-1,25276	-,17564
KNM	,2078783396	,1627126565	,14464	1,43720	,154	-,07935	,49511
KSB	,2254800810	,2705542309	,08652	2,60606	,011	,05367	,39729
A_KSB	-,0496104942	-,0346927412	,14747	-,33640	,737	-,34246	,24324
LL_Kat	-,5444136085	-,3615236175	,18077	-3,01161	,003	-,90339	-,18544
MI	-,0429021657	-,0784158191	,05169	-,83000	,409	-,14555	,05974

Dependent variable .. Ch_PDST_HP   Ch_PDST_HP: peak dorsifl ST hemi/prawa

COVARIATE	B	Beta	Std. Err.	t-Value	Sig. of t	Lower -95%	CL- Upper
DCK	-,0082008007	-,0326034194	,01951	-,42038	,675	-,04694	,03054
A_KPT	-,3234594411	-,0993023468	,25014	-1,29311	,199	-,82019	,17327
KNT	,3846462610	,1370075060	,27650	1,39110	,168	-,16444	,93373
A_KNT	1,1569245215	,2924560733	,33678	3,43521	,001	,48814	1,82571
KNM	,0826350764	,0418886936	,17962	,46006	,647	-,27405	,43932
KSB	,0762468296	,0592499822	,10744	,70965	,480	-,13711	,28961
A_KSB	,4845103364	,2194261567	,18313	2,64566	,010	,12084	,84818
LL_Kat	-,4865554575	-,2092475281	,22448	-2,16745	,033	-,93233	-,04078
MI	-,5246360069	-,6210160828	,06419	-8,17341	,000	-,65210	-,39717

Dependent variable .. A_Ch_PDSW_HP A_Ch_PDSW_HP: =Abs(Ch_PDSW_HP)

COVARIATE	B	Beta	Std. Err.	t-Value	Sig. of t	Lower -95%	CL- Upper
DCK	,0122221764	,1336836055	,00975	1,25355	,213	-,00714	,03158
A_KPT	,0742496780	,0627129120	,12502	,59390	,554	-,17401	,32251
KNT	,1005511622	,0985354836	,13820	,72760	,469	-,17388	,37498
A_KNT	-,1149710649	-,0799589135	,16832	-,68304	,496	-,44923	,21929
KNM	-,0071144467	-,0099219315	,08977	-,07925	,937	-,18538	,17115
KSB	,0086378736	,0184669849	,05370	,16086	,873	-,09800	,11527
A_KSB	-,0697290189	-,0868803609	,09153	-,76182	,448	-,25149	,11203
LL_Kat	-,0584837140	-,0691967417	,11220	-,52126	,603	-,28128	,16432
MI	-,0295885852	-,0963588627	,03208	-,92231	,359	-,09330	,03412

Dependent variable .. A_Ch_P_HP A_Ch_P_HP: =Abs(Ch_P_HP)

COVARIATE	B	Beta	Std. Err.	t-Value	Sig. of t	Lower -95%	CL- Upper
DCK	,0123297826	,0994875997	,01302	,94729	,346	-,01352	,03818
A_KPT	-,2771769273	-,1727043988	,16690	-1,66078	,100	-,60860	,05424
KNT	,0035156597	,0025415363	,18449	,01906	,985	-,36284	,36987
A_KNT	,3459738068	,1775029117	,22470	1,53969	,127	-,10024	,79219
KNM	-,1531260006	-,1575389568	,11984	-1,27774	,205	-,39111	,08485
KSB	,0202662703	,0319629538	,07169	,28271	,778	-,12209	,16262
A_KSB	,0714340078	,0656594150	,12219	,58462	,560	-,17121	,31407
LL_Kat	,1654042737	,1443714630	,14978	1,10434	,272	-,13202	,46283

COVARIATE	B	Beta	Std. Err.	t-Value	Sig. of t	Lower -95%	CL- Upper
MI	-,0108297302	-,0260177079	,04283	-,25287	,801	-,09587	,07422

Dependent variable .. Ch_SL_HP   Ch_SL_HP: step lenght hemi/prawa

COVARIATE	B	Beta	Std. Err.	t-Value	Sig. of t	Lower -95%	CL- Upper
DCK	,0000726948	,0531463905	,00014	,50366	,616	-,00021	,00036
A_KPT	,0011593369	,0654505288	,00185	,62643	,533	-,00252	,00483
KNT	-,0000701419	-,0045943539	,00205	-,03429	,973	-,00413	,00399
A_KNT	-,0010115728	-,0470236779	,00249	-,40597	,686	-,00596	,00394
KNM	,0020361430	,1898036152	,00133	1,53217	,129	-,00060	,00468
KSB	-,0005491813	-,0784776454	,00079	-,69085	,491	-,00213	,00103
A_KSB	,0008038114	,0669427186	,00135	,59324	,554	-,00189	,00349
LL_Kat	,0003921466	,0310127501	,00166	,23611	,814	-,00291	,00369
MI	,0004902189	,1067084029	,00047	1,03224	,305	-,00045	,00143

- -

* * * * * * * * * * * * * * * * * * A n a l y s i s   o f   V a r i a n c e -- Design   1 * * * * * * * * * * * * * * * * * *

EFFECT .. CONSTANT
Multivariate Tests of Significance (S = 1, M = 5 1/2, N = 39 1/2)

Test Name	Value	Exact F	Hypoth. DF	Error DF	Sig. of F
Pillais	,42947	4,69031	13,00	81,00	,000
Hotellings	,75277	4,69031	13,00	81,00	,000
Wilks	,57053	4,69031	13,00	81,00	,000
Roys	,42947				

Note.. F statistics are exact.

- -

Eigenvalues and Canonical Correlations

Root No.	Eigenvalue	Pct.	Cum. Pct.	Canon Cor.
1	,75277	100,00000	100,00000	,65534

- -

EFFECT .. CONSTANT (Cont.)
Univariate F-tests with (1;93) D. F.

Variable	Hypoth. SS	Error SS	Hypoth. MS	Error MS	F	Sig. of F
Ch_FO_ZL	923,18765	6750,00650	923,18765	72,58072	12,71946	,001
Ch_MPT_Z	1,92384	2162,05838	1,92384	23,24794	,08275	,774
A_Ch_MPR	16,52399	1049,57042	16,52399	11,28570	1,46415	,229
A_Ch_MHF	1,90346	4146,56975	1,90346	44,58677	,04269	,837

Ch_HRFE_ 47,69109 6548,42482 47,69109 70,4
1317 ,67730 ,413
A_Ch_StM 51,76290 2607,21098 51,76290 28,0
3453 1,84640 ,177
A_Ch_S_1 840,95836 9579,56253 840,95836 103,0
0605 8,16416 ,005
Ch_ICKFL 6,36544 7473,02081 6,36544 80,3
5506 ,07922 ,779
Ch_RkFlE 1689,29490 6511,20881 1689,29490 70,0
1300 24,12830 ,000
Ch_PDST_ 535,80031 10040,83221 535,80031 107,9
6594 4,96268 ,028
A_Ch_PDS 12,45496 2508,17393 12,45496 26,9
6961 ,46181 ,498
A_Ch_P_H 35,39245 4469,80250 35,39245 48,0
6239 ,73639 ,393
Ch_SL_HP ,00381 ,54964 ,00381 ,0
0591 ,64438 ,424

- -
- -

EFFECT .. CONSTANT (Cont.)
Raw discriminant function coefficients
Function No.

Variable	1
Ch_FO_ZL	,08888
Ch_MPT_Z	,01129
A_Ch_MPR	-,04573
A_Ch_MHF	-,00512
Ch_HRFE_	-,01427
A_Ch_StM	,10967
A_Ch_S_1	-,03342
Ch_ICKFL	-,02670
Ch_RkFlE	,08063
Ch_PDST_	,02843
A_Ch_PDS	-,01555
A_Ch_P_H	-,02333
Ch_SL_HP	-2,57853

- -
- -

Standardized discriminant function coefficients
Function No.

Variable	1
Ch_FO_ZL	,75723
Ch_MPT_Z	,05443
A_Ch_MPR	-,15363
A_Ch_MHF	-,03421
Ch_HRFE_	-,11974
A_Ch_StM	,58068
A_Ch_S_1	-,33920
Ch_ICKFL	-,23937
Ch_RkFlE	,67463
Ch_PDST_	,29546
A_Ch_PDS	-,08075
A_Ch_P_H	-,16173
Ch_SL_HP	-,19823

- -

Estimates of effects for canonical variables
Canonical Variable

Parameter	1
1	32,34980

- -

Correlations between DEPENDENT and canonical variables
Canonical Variable

Variable	1
Ch_FO_ZL	,42625
Ch_MPT_Z	-,03438
A_Ch_MPR	-,14462
A_Ch_MHF	-,02469
Ch_HRFE_	,09836
A_Ch_StM	,16240
A_Ch_S_1	-,34150
Ch_ICKFL	,03364
Ch_RkFlE	,58707
Ch_PDST_	,26625
A_Ch_PDS	,08122
A_Ch_P_H	-,10256

Ch_SL_HP	,09594

\- -

Abbreviated Name	Extended Name
A_Ch_MHF	A_Ch_MHF_HP
A_Ch_MPR	A_Ch_MPR_HP
A_Ch_P_H	A_Ch_P_HP
A_Ch_PDS	A_Ch_PDSW_HP
A_Ch_S_1	A_Ch_StMHR_HP
A_Ch_StM	A_Ch_StMHR_ZL
Ch_HRFE_	Ch_HRFE_ZL
Ch_ICKFL	Ch_ICKFL_ZL
Ch_MPT_Z	Ch_MPT_ZL
Ch_PDST_	Ch_PDST_HP
Ch_RkFlE	Ch_RkFlEx_HP
